# Adverse Outcomes in Neonates Following Planned Home Births: A Case Report Series and a Narrative Literature Review

**DOI:** 10.3390/jcm14041181

**Published:** 2025-02-11

**Authors:** Tommaso Bellini, Francesco Vinci, Giulia Polleri, Federico Pezzotta, Luca Grasselli, Emanuela Piccotti, Luca Antonio Ramenghi, Andrea Moscatelli, Pasquale Striano

**Affiliations:** 1 Paediatric Emergency Room and Emergency Medicine Unit, Emergency Department, IRCCS Istituto Giannina Gaslini, 16147 Genoa, Italy; tommasobellini@gaslini.org (T.B.); lucagrasselli@gaslini.org (L.G.); emanuelapiccotti@gaslini.org (E.P.); 2 Department of Neuroscience, Rehabilitation, Ophthalmology, Genetics, Maternal and Child Health, University of Genoa, 16132 Genoa, Italy; 5296194@studenti.unige.it (F.P.); lucaramenghi@gaslini.org (L.A.R.); pstriano@unige.it (P.S.); 3 Neonatal Intensive Care Unit, Department of Maternal and Neonatal Health, IRCCS Istituto Giannina Gaslini, 16147 Genoa, Italy; giuliapolleri@gaslini.org; 4 Pediatric and Neonatal Intensive Care Unit, Emergency Department, IRCCS Istituto Giannina Gaslini, 16147 Genoa, Italy; andreamoscatelli@gaslini.org; 5 Pediatric Neurology and Neuromuscular Diseases Unit, IRCCS Istituto Giannina Gaslini, 16147 Genoa, Italy

**Keywords:** early-onset neonatal sepsis, hypoglycemia, home births, kernicterus, morbility, pediatric emergency department

## Abstract

**Background:** Although home births provide personal and intimate experiences, they pose potential risks that may be better managed in hospital settings. The safety of home birth remains highly debated, with no consensus on its safety or potential adverse events, and its adoption varies widely across the world. In Italy, the Italian Society of Neonatology opposed this practice, resulting in one of the lowest home birth rates in Europe (approximately 0.1% of total births). This study evaluated the impact of planned home births on neonatal health, with a focus on severe complications requiring intervention in the pediatric emergency department (PED). **Methods:** Cases were collected from patients admitted to IRCCS Istituto Giannina Gaslini Children’s Hospital between January 2022 and December 2024. The analysis focused on neonates born at home, who required emergency care for life-threatening conditions. **Results:** We identified five cases, with an incidence of approximately 0.65 per 10,000 PED visits and a complication rate of 15–30% for all planned home births. Factors contributing to unfavorable outcomes include lack of advanced medical equipment, timely diagnostics, and comprehensive postpartum screening. Thus, while proponents argue that home births can provide a more comfortable and personalized experience, concerns about safety and associated risks persist. **Conclusions:** This study aims to highlight the necessity of adopting hospital-level neonatal care protocols for home births, particularly during the critical first 48 h of life, to mitigate risks and ensure optimal neonatal outcomes.

## 1. Introduction

Place of delivery has consistently been found to be associated with maternal and neonatal outcomes [1]. Thus, the global trend of home births has elicited considerable discussion among healthcare professionals, particularly regarding the potential hazards that may arise from this practice [2,3]. Although home births may provide an intimate and convenient environment for certain mothers, they may also expose both themselves and newborns to a variety of preventable complications that are typically managed more effectively in hospital settings [2,4]. Thus, previous studies found that neonates born at institutional health facilities have a lower risk of death compared to neonates who were born at home [5]. However, emotion, debate, and controversy surrounding the topic of home birth are clearly presented, with studies both supporting and against this practice [2,3,4,6,7]. Home births may be influenced by various factors and parental choices. In underserved or rural areas, the scarcity of maternity care services poses significant challenges for pregnant women, creating barriers to timely and adequate care [3]. Geographic isolation further exacerbates this issue, as women residing in remote locations may face difficulties in reaching healthcare facilities during labor. In cases of precipitous delivery, this delay can result in an unplanned home birth with minimal or no medical assistance [2,3,4,5,8]. Additionally, cultural preferences and the growing mistrust of medical institutions have contributed to an increase in planned home births. These planned events are often driven by a desire to avoid hospitalization and embrace a perceived sense of autonomy and comfort associated with the domestic environment or midwifery care [2,3]. However, home birth scenarios can potentially delay access to specialized medical interventions during critical moments, thereby increasing the likelihood of adverse maternal and neonatal outcomes. For instance, it has clearly indicated that delivery outside a health facility is more likely to lead to neonatal death compared with delivery in a health facility; this confirms the role of place of delivery on newborn survival [8].

A notable concern is the increased risk of neonatal complications associated with home birth [7,9]. Such complications may include neonatal respiratory distress, hypothermia, and infections, which may require immediate and advanced medical intervention. Consequently, delays in access to emergency care can lead to preventable morbidity and mortality. Furthermore, postpartum hemorrhage and retained placenta are significant maternal risks that may also arise during home births, necessitating rapid and often complex medical interventions that are unavailable outside hospital settings. Beyond immediate physical risks, home birth can have long-term implications for neonatal development and maternal mental health, particularly in cases where adverse events occur [7]. For these reasons, countries such as the Netherlands, New Zealand, and the UK have guidelines that indicate that home birth should be offered to low-risk women only.

In our analysis of recent cases and the pertinent literature, we examined the emergence of novel clinical entities linked to home births that are not typically encountered in pediatric emergency departments (PEDs). These conditions are frequently observed within the first few days of a newborn’s life and are often identified and managed during inpatient stays in Maternity and Neonatal Assistance Units or Neonatal Intensive Care Units (NICUs). For instance, undiagnosed congenital anomalies, untreated neonatal jaundice, and inadequate thermal regulation may escalate in severity owing to delayed diagnosis and intervention in home birth settings. Our study aimed to evaluate the impact of home birth complications potentially managed in a PED because this shift in the point of care underscores the critical need for pediatric emergency physicians to be equipped with comprehensive knowledge in neonatology. Such expertise is essential to effectively address and manage acute complications resulting from home births, particularly because this practice continues to gain traction globally [2,3,4,5,7,8,10,11].

## 2. Materials and Methods

This case series was collected by analyzing admissions at the PED of the IRCCS Istituto Giannina Gaslini Children’s Hospital, a highly specialized third-level pediatric hospital located in Genoa within the Liguria Region of Italy. This institution is renowned for its excellence in pediatric care, offering a comprehensive approach to pediatric health, and serving as a critical resource for children and their families from Italy and other countries.

To investigate the implications of home births on neonatal health outcomes, we systematically collected data on all home-born neonates admitted to the PED for acute clinical issues. The case series period spanned from 1 January 2022, to 31 December 2024, allowing us to gather a substantial dataset that reflects both seasonal variations and the evolving landscape of home birth practices over time. We then compared these patients to the total number of PED visits, total number of regional births, and the total number of planned home births in the period considered. This timeframe also allowed us to capture trends influenced by external factors, such as public health campaigns, policy changes, and shifts in parental preferences regarding home births. These contextual factors were analyzed in conjunction with clinical data to provide a more comprehensive understanding of the issue.

During the study period, we documented the demographic information, clinical presentations, therapies administered, and outcomes of each neonate. We also assessed the specific circumstances surrounding their births, including the presence or absence of trained medical personnel, and any immediate postnatal interventions that may have been provided. This comprehensive data collection aimed to provide insights into the types of complications that arise from home births and the subsequent management of these cases in a pediatric emergency setting.

Patients admitted to the PED of home-born neonates beyond 72 h of life were excluded from this study. This exclusion criterion was carefully established to focus on the acute complications typically associated with the immediate neonatal period. By narrowing the scope to early neonatal admissions, we aimed to identify the patterns and risk factors unique to the transitional period following birth. This period is crucial for identifying conditions that can lead to significant morbility and mortality if left untreated. Through this case series and thorough review of the literature, we aim to contribute to the growing body of evidence on neonatal risks associated with home births and provide actionable insights for improving neonatal emergency care protocols.

A systematic literature search was conducted using *PubMed* to identify relevant articles. Two authors (T.B., F.V.) independently conducted the search process to ensure comprehensive coverage. Keywords included “Home-Birth”, “Out-of-Hospital Birth”, and “Neonatal Outcome” and focused on articles published in the last 10 years. Search terms were selected based on their relevance to the research question and were combined using Boolean operators. Filters were used to limit the search to articles written in English and published in peer-reviewed journals. Additionally, manual searches were performed by screening the reference lists of the relevant articles. Duplicates were identified and removed using reference management software (Zotero v6.0.26, Corporation for Digital Scholarship, Vienna, VA, USA), and any discrepancies in article selection were resolved through consensus. The final selection of articles was based on their relevance to the research question and quality of evidence presented. Data extraction was performed independently by two reviewers and crosschecked to ensure accuracy.

## 3. Results and Case Description

We identified a total of five patients who were admitted to the PED due to complications arising from home births. Notably, all home births were planned, and we did not identify any cases of home-born neonates who presented to the PED beyond 72 h of life; therefore, no patients were excluded from our analysis. This indicates that all the identified cases were within the critical neonatal period. Furthermore, considering approximately 38,000 visits per year to our PED, the incidence of planned home birth-related admissions to the PED was calculated to be 0.65 per 10,000 PED visits. Given the rarity of the event and the limited case series involving only five patients, we provide a brief description of each clinical case below.

### 3.1. Case 1

This term newborn was delivered at home via Lotus birth after an uncomplicated pregnancy [12]. Delivery occurred without professional medical supervision, reflecting the family’s preference for a natural birth experience. However, concerns arose when the infant was brought to the PED at 72 h of life due to decreased reactivity, poor feeding, and significant jaundice. The umbilical cord was excised using non-sterile scissors on the second day of life, which increased the risk of infection. Upon clinical evaluation, the infant was found to have severe hyperbilirubinemia, with bilirubin levels reaching 51 mg/dL, which is a critical threshold associated with neurotoxicity [13]. Additionally, the C-reactive protein levels were markedly elevated, suggesting systemic inflammation.

The newborn was admitted to the NICU and underwent urgent treatment, including multiple exchange transfusions, to address the dangerously high bilirubin levels. Blood cultures identified a polymicrobial infection involving Enterococcus faecalis, Staphylococcus aureus, and Escherichia coli, which was managed with aggressive broad-spectrum antibiotics. Further investigations revealed maternal–fetal ABO incompatibility, which likely contributed to the severity of hyperbilirubinemia [13].

At 20 days of life, advanced imaging studies, including brain Magnetic Resonance Imaging (MRI), identified bilateral hyperintensities in the hippocampi and subthalamic nuclei, consistent with kernicterus. These results underscore the profound neurological impact of untreated hyperbilirubinemia and delayed interventions. Neonatal hyperbilirubinemia is a common phenomenon and, in most cases, is benign and transient. However, in severe cases, neonates can develop encephalopathy and kernicterus, and prompt management is mandatory. With appropriate screening and treatment, the adverse sequelae can be prevented [14]. This case highlights the importance of sterile practices, the early detection of jaundice, and timely access to specialized neonatal care to prevent severe outcomes [13,15].

### 3.2. Case 2

This was a term newborn delivered via spontaneous vaginal delivery with intrauterine growth restriction (IUGR) and meconium-stained amniotic fluid. The infant was brought to the PED at 36 h of life due to poor reactivity and difficulty feeding at home, where she had been fed formula milk. The birth weight was 2090 g, classified as a 1st-centile, small for gestational age (SGA) infant according to the INeS charts [16]. Upon arrival at the PED, the neonate was in a critical condition, pale, and unresponsive, necessitating immediate medical intervention. Blood tests revealed no detectable blood glucose levels, indicative of severe neonatal hypoglycemia.

The patient was promptly treated with an initial intravenous (IV) bolus of glucose, followed by a continuous infusion of 10% glucose, which restored blood glucose to 33 mg/dL within 30 min. A second glucose bolus was required to stabilize the infant’s metabolic state, which led to clinical improvement. Despite this, the severity of the hypoglycemic episode had already caused significant neurological damage. On the third day of life, brain MRI revealed extensive bilateral occipitoparietal cortical damage, which was attributed to probable hypoglycemic encephalopathy. Follow-up imaging at one year of age showed progression to gliotic and malacic changes, indicating chronic sequelae of the initial injury [17].

This case underscores the importance of the early recognition and prompt management of neonatal hypoglycemia, especially in at-risk infants such as those with IUGR or SGA. It also highlights the critical need for close monitoring and follow-up care to address potential long-term neurological impairment.

### 3.3. Case 3

This term newborn was delivered at home via spontaneous vaginal delivery and was brought to the emergency department at 36 h of life due to poor feeding and lethargy observed over the preceding 12 h. The delivery was performed without professional medical supervision, and the umbilical cord was excised using an antiseptic solution without sterile tools, raising concerns about the risk of infection. Upon arrival at the PED, the infant exhibited significant clinical symptoms including hypoactivity, hypotonia, pallor, and intermittent episodes of apnea and desaturation. The high-pitched crying and intermittent consolability further underscored the severity of the clinical presentation.

No detailed information was available regarding maternal Group B Streptococcus (GBS) colonization status or the administration of intrapartum antibiotic prophylaxis, complicating the clinical assessment [15]. The neonate was promptly admitted to the NICU, where empiric broad-spectrum antibiotic therapy was initiated for suspected early onset neonatal sepsis (EONS) [15]. Blood culture results confirmed the presence of methicillin-sensitive Staphylococcus aureus (MSSA), validating the initial clinical suspicion. The infant was also found to have polycythemia, a condition that requires careful monitoring and management during the NICU stay.

Over the two-week hospitalization, the newborn responded well to treatment, including antibiotics and supportive care. No additional complications occurred, and the patient demonstrated complete clinical recovery. A follow-up evaluation at 12 months of life revealed normal development with no evidence of long-term sequelae, emphasizing the critical role of early detection and intervention in mitigating adverse outcomes associated with home deliveries. This case highlights the importance of proper infection control measures, intrapartum risk assessments, and timely medical attention in the management of home-born neonates presenting with sepsis-like symptoms [15].

### 3.4. Case 4

This case report describes a term newborn with IUGR delivered at home via spontaneous vaginal delivery, who presented to the emergency department on the third day of life with lethargy, restlessness, poor feeding, and weak suckling. On examination, the infant was noted to be irritable and hypotonic, with clinical signs of dehydration, including pallor, tachycardia, sunken anterior fontanelle, and sunken eyes. These findings were suggestive of significant fluid imbalance and metabolic disturbances [18,19].

Initial laboratory tests confirmed severe hypernatremia with a sodium level of 171 mEq/L, while other blood parameters remained within normal limits. The neonate’s critical condition necessitated immediate intervention to stabilize electrolyte imbalance and restore hemodynamic function. The infant initially received two boluses of 0.9% saline to correct hypovolemia and improve circulation. Subsequently, the neonate was admitted to the NICU for continuous management, including IV with 0.45% hypotonic saline [18,19].

Sodium levels gradually normalized by the third day of therapy, with careful monitoring to avoid rapid correction, which could precipitate cerebral edema. A subsequent brain MRI was performed to assess the potential complications of hypernatremia. Imaging ruled out cerebral hemorrhage and venous sinus thrombosis, which are associated with severe electrolyte disturbances. The absence of such complications was a positive prognostic indicator.

The infant showed steady clinical improvement and was discharged after a thorough evaluation. Follow-up assessments revealed no developmental delay or neurological sequelae. This case highlights the importance of the early recognition and management of hypernatremia, especially in home-delivered infants, who may be at increased risk due to inadequate feeding practices and delayed access to healthcare [18,19].

### 3.5. Case 5

A male neonate was born at home following uncomplicated eutocic delivery. The family chose to give birth at home and did not receive any obstetric support. Six hours after birth, the infant was found to be unresponsive by the grandmother, who promptly contacted emergency territorial services. On arrival, the neonate exhibited severe hypothermia, with a core temperature of 32 °C, and bradycardia, with a heart rate of 70 bpm. The monitoring of vital signs revealed recurrent episodes of apnea and desaturation, which further compromised the infant’s condition. The emergency physician performed orotracheal intubation and external cardiac massage to stabilize the neonate before transport to the PED.

Upon arrival at the NICU, the neonate was placed in an incubator for controlled thermal management and was rehydrated with intravenous fluids. Gradual warming restored the infant’s normal skin temperature within a few hours, and vital signs stabilized. Comprehensive diagnostic evaluations, including blood tests and imaging, were performed to assess the underlying conditions or complications associated with hypothermia [20].

Brain MRI scans performed on the third and fifteenth days of life showed no evidence of cerebral hemorrhage, ischemia, or venous sinus thrombosis, which are potential complications of severe hypothermia. The neonate’s clinical condition steadily improved, and no additional interventions were required during the NICU stay. The infant was discharged in good health after a thorough evaluation, with follow-up visits confirming normal developmental milestones. Prevention of hypothermia in the delivery room is a cost-effective, high-impact intervention to reduce neonatal mortality, especially in preterm neonates [20]. This case underscores the risks associated with unassisted home births and the importance of early detection and prompt medical intervention to prevent long-term sequelae from conditions such as neonatal hypothermia.

## 4. Discussion

Throughout human history, childbirth has almost universally taken place in the home environment, where midwives, relatives, or other trusted community members were the primary caregivers assisting in the process [21]. This tradition has remained largely unchallenged for centuries, as home births have been considered the norm. However, following World War II, there was a significant societal shift in Italy and other parts of the Western world towards hospital-based deliveries. This transition became particularly prominent in the 1960s and the 1970s [21,22,23,24,25]. Several factors played a role in this change, including advancements in medical technology and hospital infrastructure, evolving healthcare policies aimed at ensuring the safety of both mother and child, and broader cultural and societal transformations that emphasized the reliability of institutionalized care [2,7,22,23,25,26]. By the end of the 1970s, hospital births had become standard practice across Italy and other developed nations, accompanied by a notable decline in neonatal mortality rates, which can be directly attributed to improved access to professional medical interventions [2,9,13,21,22,23].

In recent years, there has been a resurgence in interest in home births in various developed countries. This renewed enthusiasm is often driven by personal, cultural, or ideological beliefs, reflecting a desire for more natural or individualized birthing experiences [2,3,21,22,25,26,27]. Despite this trend, Italy maintains one of the lowest rates of out-of-hospital deliveries in Europe, with home births estimated to account for only approximately 0.1–0.2%, less than the United States or other European countries where 0.5% of mothers give birth in freestanding birth centers [7,22,23,28].

While precise data on the regional distribution of home births remain unavailable, estimates suggest that in Liguria, where approximately 8000 births occur annually, planned home deliveries range from 8 to 16 cases per year. Thus, our five cases may represent a complication rate of approximately 15–30% of all Ligurian home births, which is in line with a recent report by a Cochrane meta-analysis [10,11]. Furthermore, the clinical cases described corresponded to an incidence of 0.62 per 10,000 visits to the ER, representing a rare but not negligible event. However, according to other authors, there are insufficient data to evaluate the levels of maternal mortality and severe morbidity according to the place of birth [7,9].

Many parents choose home births to circumvent the perceived medicalization of childbirth and desire a more natural and personal experience [25,27]. Some studies have suggested that planned home deliveries attended by qualified midwives and low-risk pregnancies can be as safe as hospital births under specific circumstances, even with a lower need for intervention [23,25,29,30,31]. However, the safety of home births remains a highly debated and controversial topic, especially when unforeseen complications arise or when risk factors are not adequately identified before labor, and the potential risks to newborns cannot be overlooked [2,3,4,7,9,15,21,22,23,25,26,29,31,32]. Thus, the Italian Society of Neonatology reiterating its opposition to this practice states that the possibility of neonatal mortality in the case of home birth is 2.6 times greater than that in a hospital birth [33]. This statement, however, appears to be in contrast with some local Italian realities where local health authorities propose home birth or maternity home programming after careful evaluation of the pregnant woman and proven low risk [34,35,36]. Local health authorities point out that the organization of home births, and the choice of which ones can take place at home, must be completely seamless and risk-free by ensuring access to trained medical personnel and emergency protocols. These should include pre-delivery risk assessments, coordination between home birth professionals and healthcare facilities, immediate postpartum monitoring, and early clinical checks scheduled by trained pediatricians for each home birth.

Education campaigns that emphasize the importance of risk assessment, contingency planning, and timely hospital transfers can further mitigate risks. Developing protocols to guide emergency responses in cases of planned or unplanned home births is also essential. Ultimately, our case series highlights the necessity of bridging the gap between home birth practices and the current healthcare system to mitigate risks and optimize neonatal outcomes, while respecting parental autonomy and cultural preferences.

Despite the limited number of cases, each of them provided valuable insights into the challenges and risks associated with planned home birth. Clinical presentations vary significantly, ranging from respiratory distress and neonatal sepsis to hypothermia. In all cases, delays in medical intervention were attributed to logistical challenges such as the lack of immediate access to advanced neonatal resuscitation or the delayed recognition of complications by non-medical birth attendants. The presence of skilled personnel during these planned home births was inconsistent, with some cases involving certified midwives and others relying solely on informal support systems. This variability highlights the critical role of trained professionals in ensuring safe patient outcomes.

Our case series underscores the multifaceted risks associated with non-traditional birthing practices, particularly when compounded by improper handling of the umbilical cord, failure to address known risk factors, and delayed access to medical care. Case 1 exemplifies the severe neurological consequences of untreated hyperbilirubinemia with kernicterus serving as a stark reminder of the importance of timely recognition and intervention [13,14]. The bilateral hyperintensities observed in the hippocampi and subthalamic nuclei highlight the characteristic damage induced by elevated bilirubin levels [14]. Case 2 reveals the dangers of untreated neonatal hypoglycemia, particularly in neonates with IUGR and those classified as SGA [17]. Progression to bilateral occipitoparietal damage, as evidenced by MRI findings, and subsequent gliotic evolution emphasizes the long-term implications of hypoglycemic encephalopathy [16].

Case 3 focused on infection prevention, where MSSA sepsis resulted from non-sterile cord care practices and the absence of antibiotic prophylaxis [15]. These represent modifiable risk factors that can significantly improve outcomes, as perinatal asphyxia, meconium contamination in amniotic fluid, GBS colonization in pregnant women, chorioamnionitis, the premature rupture of membranes, lower gestational age, maternal urinary tract or reproductive tract infection, perinatal fever, and very low birth weight are preventivable risk factors that may increase the risk of EONS ≥3 times [4,12,15]. Case 4 highlights the critical dangers of dehydration and hypernatremia due to inadequate fluid intake, emphasizing the essential need for the careful monitoring of feeding and hydration in home birth scenarios [15,18,19]. Fortunately, no long-term adverse effects were observed in Cases 3 and 4. Case 5 underscores the critical importance of adequate perinatal care, particularly in unassisted home deliveries, as the early identification of neonatal conditions, such as hypothermia and bradycardia, is paramount to avoid serious complications [19]. Fortunately, this neonate also experienced no long-term sequelae.

Collectively, these cases underscore the necessity for rigorous monitoring, thorough risk assessment, and early intervention, particularly in neonates with additional risk factors [13,18,19]. All these pathologic scenarios were detected by clinical control performed within 48 h of life, suggesting that these controls must be scheduled for each home birth in this timeframe. The need to ensure the safety, quality, and appropriateness of home births is incontrovertible. The risk profile associated with home deliveries is profoundly influenced by the availability of timely medical interventions, which are often limited in out-of-hospital settings [18,19]. Additionally, this analysis revealed that immediate postnatal interventions, such as thermoregulation and the early detection of hypoglycemia and jandice, are often inadequate in-home settings [13,14,17,20]. This underscores the importance of structured guidelines and contingency planning for home births.

The findings from these cases aim to inform future strategies to bridge the gap between home birth practices and the healthcare system, to optimize neonatal outcomes and reduce preventable complications. As our case series and the existing literature highlight, the delayed diagnosis and treatment of preventivable, critical neonatal conditions frequently lead to increased morbidity and mortality rates [2,3,7,13,20,21,25,30,32].

Although home births may be safe under specific, carefully controlled circumstances, the cases presented reveal that incomplete perinatal histories, the absence of advanced neonatal resuscitation tools, delays in performing diagnostic blood tests and imaging, and the time required to transfer a critically ill neonate to a hospital can result in unfavorable outcomes [2,3,5,8,9,14,15,29,30,31,32,37]. Even in apparently uncomplicated deliveries, home-born neonates remain at risk of missing vital postpartum checks, including Coombs testing and monitoring for hyperbilirubinemia, hypoglycemia, weight loss, and other metabolic or cardiological issues. Essential screenings, such as audiological and metabolic assessments, are conducted more reliably in hospital environments. Alarmingly, out-of-hospital births are often associated with higher rates of refusal for neonatal prophylaxis and screening programs, further compounding the risks [38,39].

Finally, in a hospital setting, the presence of highly trained healthcare professionals, coupled with access to advanced medical equipment and technologies, ensures the prompt detection and effective management of neonatal complications. This environment significantly reduces the risks associated with delays in diagnosis and treatment. By contrast, home birth settings often lack these critical resources, meaning that even minor delays in recognizing and addressing complications can lead to serious or potentially life-threatening outcomes in neonates. To better prepare healthcare providers and PED physicians for these scenarios, Table 1 highlights the key conditions that they should be equipped to address when managing neonates delivered in a home birth context.

Our study had some limitations. It was a retrospective study, which inherently carries the potential for biases, such as incomplete data capture, recall bias, and lack of randomization. Additionally, the sample size was insufficient to draw statistically significant conclusions, limiting the generalizability of our findings. Future research should focus on conducting larger, multicenter prospective studies and meta-analyses to comprehensively address this topic and provide more robust evidence.

## 5. Conclusions

The rising trend of home births highlights critical gaps in neonatal care, particularly during the vulnerable first 24 h–48 h of life, a period often described as the most crucial for a newborn’s survival and long-term health. Although home births may be considered safe for low-risk pregnancies attended by qualified professionals, they inherently carry significant risks that can escalate quickly without immediate access to advanced medical interventions. Issues such as respiratory distress, infections, dehydration, hypoglycemia, hyperbilirubinemia, or undiagnosed congenital anomalies may emerge suddenly, requiring rapid medical attention that is readily available in the hospital setting. Therefore, if home births are chosen and approved by local health authorities, public health institutions should always be involved in and support safe home birth practices. PEDs may play a pivotal role in bridging this gap, as they are often the first point of contact for managing acute complications in home-born neonates before they are transferred to specialized NICUs. These departments must be equipped with appropriate resources, trained personnel, and clear protocols to address emergencies efficiently. These measures are essential to ensure the safety, well-being, and equitable care of newborns, regardless of their birthing location or circumstances, reduce disparities, and foster better outcomes for newborns in diverse environments. In conclusion, as the trend towards home births continues, it is imperative that healthcare providers ensure that women are well informed about the potential risks and benefits, promoting a careful selection process for candidates considering this option.

## Figures and Tables

**Table 1:** Main neonatal clinical entities that the emergency pediatrician must get used to managing. Modified from [3,6,8,9,10,11,14,16,17,18,20,22,23].

Main Clinical, Critical Neonatal Diseases
Increased Neonatal Infectious Risk Possible Early-Onset Neonatal Sepsis Missed maternal serologies Hypoglycemia Respiratory Distress Syndrome Neonatal asphyxia Hypothermia Dehydratation Hypernatriemia Polycythemia Kernicterus Neonatal Seizures Diseases due to screening and prophylaxis not performed Hemolytic disease of the newborn Missed Cardiac Defects Missed inborn errors of metabolism

## Data Availability

The original contributions presented in this study are included in the article. The datasets used and analyzed in this study are available from the corresponding author upon reasonable request.
